# Photosynthetic production of glutamine through metabolic analysis-based engineering of *Picosynechococcus* sp. PCC 7002

**DOI:** 10.1016/j.mec.2026.e00284

**Published:** 2026-06-17

**Authors:** Yuichi Kato, Ayaka Tsuji, Yuji Haraguchi, Tatsuya Shimizu, Akihiko Kondo, Tomohisa Hasunuma

**Affiliations:** a Engineering Biology Research Center, Kobe University, 1-1 Rokkodai, Nada, Kobe, 657-8501, Japan; b Graduate School of Science, Technology and Innovation, Kobe University, 1-1 Rokkodai, Nada, Kobe, 657-8501, Japan; c Institute of Advanced Biomedical Engineering and Science, TWIns, Tokyo Women's Medical University, 8-1 Kawada, Shinjuku, Tokyo, 162-8666, Japan; d Department of Chemical Science and Engineering, Faculty of Engineering, Kobe University, 1-1 Rokkodai, Nada, Kobe, 657-8501, Japan; e RIKEN Center for Sustainable Resource Science, 1-7-22 Suehiro, Tsurumi, Yokohama, Kanagawa, 230-0045, Japan

**Keywords:** Cyanobacteria, Glutamine, Metabolic analysis, *Picosynechococcus* sp. PCC 7002

## Abstract

Glutamine is an important nitrogen donor in the biosynthesis of nucleotides and several other amino acids. Proliferating cells consume high amounts of glutamine, and cell culture media contain glutamine as the most abundant amino acid. Glutamine is industrially manufactured through bacterial fermentation, which requires external supplementation with sugars as the carbon source. Using the cyanobacterium *Picosynechococcus* sp. PCC 7002, this study aimed to develop a method for the photosynthetic production of glutamine using CO_2_ as the sole carbon source. The introduction of glutamate dehydrogenase from *Corynebacterium glutamicum* and glutamine synthase from *Saccharomyces cerevisiae* increased the concentration of extracellularly released glutamine. Metabolome analysis revealed decreased intracellular citrate levels in glutamine-producing cells. To enhance citrate replenishment, metabolic engineering approaches, including l-lactate assimilation and glycogen deficiency, were examined. The introduction of pyruvate carboxylase and citrate synthase from *C. glutamicum* significantly increased glutamine production. After optimizing light intensity and CO_2_ concentration, the recombinant strain produced 1168.5 μM (170.76 mg L^−1^) glutamine. This study establishes metabolic engineering approaches for converting CO_2_ into glutamine and demonstrates that cyanobacteria are promising photosynthetic producers of glutamine.

## Highlights

• Glutamine-producing cyanobacteria were developed through genetic engineering.
• Heterogeneous glutamate dehydrogenase and glutamine synthase were introduced.
• Citrate replenishment was suggested as the bottleneck for glutamine production.
• Introduction of pyruvate carboxylase and citrate synthase increased glutamine titers.
• Cyanobacteria were suggested as potential photosynthetic producers of glutamine.

## Graphical abstract

**Figure:**
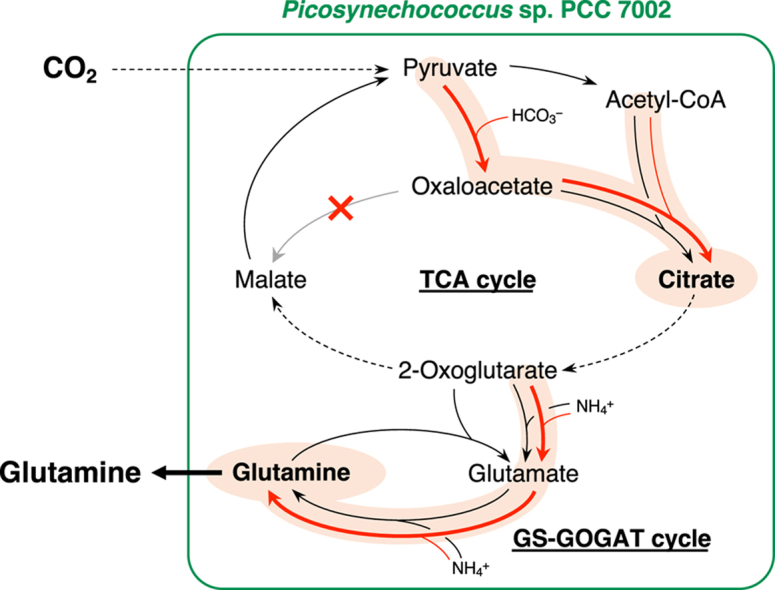

## 1 Introduction

Glutamine, a non-essential amino acid in mammals, is an important donor of nitrogen and carbon for the biosynthesis of purine and pyrimidine bases, together with other amino acids such as glutamate and asparagine (Zhang et al., 2017; Yoo et al., 2020). Proliferating cells consume high amounts of glutamine, which is the most abundant amino acid in mammalian plasma and conventional cell culture media (Ackermann and Tardito, 2019). Recently, the industrial utilization of animal cell culture has been extensively studied for the production of biopharmaceuticals and cultured meats, anticipating that the demand for glutamine will increase in the near future (Wurm, 2004; Post, 2014). Glutamine is industrially manufactured through bacterial fermentation using organisms such as *Corynebacterium glutamicum*; however, this method requires external supplementation of nutrients, especially sugars, as the carbon source (Kusumoto, 2001).

Glutamine is biosynthesized through glycolysis, the tricarboxylic acid (TCA) cycle, and the glutamine synthase (GS)-glutamate synthase (GOGAT) cycle (Liu et al., 2021; Zhang and Bryant, 2011). The gateway enzyme of the TCA cycle is citrate synthase (CS), which uses acetyl-coenzyme A (acetyl-CoA) and oxaloacetate as substrates. Oxaloacetate is supplied through the TCA cycle and anaplerotic pathways using phosphoenolpyruvate carboxylase (PEPC) and pyruvate carboxylase (PYC) (Xu et al., 2018). In *C. glutamicum*, PYC has been reported to be a major rate-limiting enzyme in the production of amino acids, and its overexpression results in the increased production of glutamate and lysine (Peters-Wendisch et al., 2001). In the GS-GOGAT cycle, bacterial GS is inactivated through reversible adenylylation in the presence of excess ammonium (Merrick and Edwards, 1995; Jakoby et al., 1999; Rehm et al., 2010). A previous study reported that knockout of GS adenylyltransferase and overexpression of GS from *Saccharomyces cerevisiae* improved glutamine production in *C. glutamicum*, achieving a titer of 73.5 g L^−1^ (Lv et al., 2021). Similarly, another study found that optimization of the GS-GOGAT cycle in *C. glutamicum* by overexpressing GS genes from *Bacillus subtilis* and *Lactobacillus acidophilus*, together with knockout of the GOGAT and glutaminase genes, resulted in glutamine production of 98.7 g L^−1^ (Liu et al., 2021). In addition to the GS-GOGAT cycle, glutamine can also be synthesized from 2-oxoglutarate and ammonia by glutamate dehydrogenase (GDH).

Cyanobacteria are prokaryotic microorganisms that can perform oxygen photosynthesis. As they can utilize CO_2_ as a carbon source, cyanobacteria have been studied as inexpensive producers of valuable organic compounds, including amino acids (Knoot et al., 2018; Kato et al., 2022). The marine cyanobacterium *Picosynechococcus* sp. PCC 7002 (PCC 7002), which expresses a feedback-resistant aspartate kinase from *Xenorhabdus bovienii* and a lysine exporter from *E. coli*, produces lysine via secretion, which is further enhanced by expressing PEPC, PYC, and feedback-resistant dihydrodipicolinate synthase from *C. glutamicum* (Korosh et al., 2017; Wang et al., 2025). The fast-growing *Synechococcus elongatus* UTEX 2973 (UTEX 2973) has been shown to produce 556.3 mg L^−1^ lysine by expressing the feedback-resistant aspartate kinase and lysine exporter genes from *E. coli* (Dookeran and Nielsen, 2021). *Synechocystis* sp. PCC 6803 (PCC 6803), expressing feedback-resistant 3-deoxy-d-arabino-heptulosonate-7-phosphate synthase and chorismate mutase/prephanate dehydrogenase genes from *E. coli*, has been shown to produce aromatic amino acids, particularly phenylalanine (Brey et al., 2020). Additionally, PCC 6803 expressing feedback-resistant 3-deoxy-d-arabino-heptulosonate-7-phosphate synthase and anthranilate synthase has been shown to produce tryptophan via secretion (Deshpande et al., 2020). PCC 6803 expressing a glutamate exporter gene from *E. coli* produced 2.3 g L^−1^ glutamate (Matsudaira et al., 2020). Furthermore, the glycogen-deficient Δ*glgC* strain of *Synechococcus elongatus* PCC7942 (PCC 7942) has been shown to secrete various amino acids, especially glutamate (Kato et al., 2024, 2025). These reports suggest the potential of cyanobacteria for amino acid production.

**Fig. 1:**
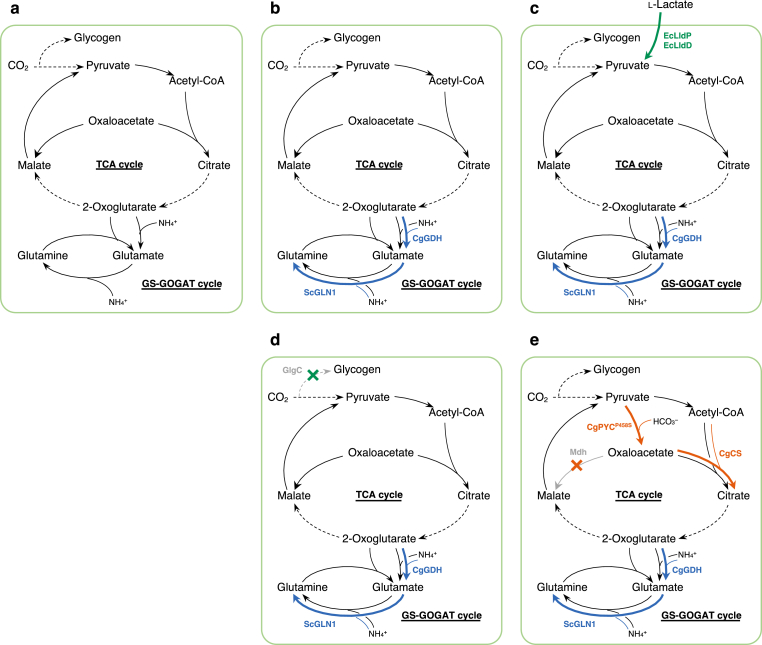
Metabolic pathway modifications performed in this study. (a) Metabolic pathway of wild-type PCC 7002. (b) Metabolic pathway of KC0111 expressing CgGDH and ScGLN1. (c) Metabolic pathway of KC0112 expressing EcLldP and EcLldD. (d) Metabolic pathway of KC0136 deficient in the *glgC* gene. (e) Metabolic pathway of KC0157 deficient in the *mdh* gene and expressing CgPYC^P458S^ and CgCS.

This study aimed to achieve the photosynthetic production of glutamine using PCC 7002 as a genetically engineered host. Based on the aforementioned studies (Lv et al., 2021; Liu et al., 2021), the present study attempted to reinforce GS activity by introducing a GS gene from *S. cerevisiae* (*ScGLN1*) (Fig. 1). The GDH gene from *C*. *glutamicum* (*CgGDH*) was also introduced to enhance glutamine synthesis from 2-oxoglutarate. Although wild-type PCC 7002 secreted little glutamine, the introduction of the *CgGDH* and *ScGLN1* genes led to glutamine production via secretion. This recombinant strain was subjected to metabolic analysis, a powerful tool in synthetic biology, to derive further genetic engineering approaches for improving glutamine production (Kato et al., 2024). As the recombinant strain exhibited decreased intracellular citrate levels, this study identified citrate replenishment as a potential bottleneck for glutamine production. Thus, several engineering approaches to enhance the TCA cycle were examined in glutamine-producing cyanobacteria. Notably, cyanobacteria harboring *C. glutamicum* PYC^P458S^ (CgPYC^P458S^) and CS (CgCS) showed an 8.88-fold increase in glutamine production compared with the parent strain. By optimizing light intensity and CO_2_ concentration, a maximum glutamine production of 1168.5 μM (170.76 mg L^−1^) was achieved through photosynthesis. This study developed metabolic engineering approaches for converting CO_2_ into glutamine and showed that cyanobacteria are promising photosynthetic producers of glutamine.

## 2 Materials and methods

### 2.1 Culture conditions

The cyanobacterium *Picosynechococcus* sp. PCC 7002 and the recombinant strains used in this study are listed in Table 1. Cyanobacteria were cultured in double-deck flasks on a BR-40LF bioshaker (TAITEC, Aichi, Japan) at 30 °C. The upper stage of the flasks was supplemented with 70 mL of medium, and the lower stage was supplemented with 50 mL of a 2 M K_2_CO_3_/KHCO_3_ solution to adjust the CO_2_ gas concentration in the flask (Kato et al., 2023, 2024). Cyanobacteria were cultured using Medium A2 (8.30 × 10^−3^ M tris(hydroxymethyl)aminomethane, 1.76 × 10^−2^ M NaNO_3_, 3.10 × 10^−1^ M NaCl, 2.00 × 10^−2^ M MgSO_4_·7H_2_O, 2.50 × 10^−3^ M CaCl_2_·2H_2_O, 3.70 × 10^−4^ M KH_2_PO_4_, 8.10 × 10^−3^ M KCl, 8.90 × 10^−5^ M Na_2_EDTA·2H_2_O, 3.00 × 10^−5^ M FeCl_3_·6H_2_O, 5.50 × 10^−4^ M H_3_BO_3_, 2.20 × 10^−5^ M MnCl_2_·4H_2_O, 2.30 × 10^−6^ M ZnCl_2_, 2.10 × 10^−7^ M Na_2_MoO_4_·2H_2_O, 1.20 × 10^−8^ M CuSO_4_·5H_2_O, 5.10 × 10^−8^ M CoCl_2_·6H_2_O, and 3.00 × 10^−9^ M vitamin B_12_) (Ludwig and Bryant, 2011; Hasunuma et al., 2019). To examine the culture conditions, MAD2 medium (8.30 × 10^−3^ M tris(hydroxymethyl)aminomethane, 1.76 × 10^−1^ M NaNO_3_, 3.10 × 10^−1^ M NaCl, 2.00 × 10^−2^ M MgSO_4_·7H_2_O, 2.50 × 10^−3^ M CaCl_2_·2H_2_O, 2.05 × 10^−3^ M KH_2_PO_4_, 8.10 × 10^−3^ M KCl, 8.90 × 10^−5^ M Na_2_EDTA·2H_2_O, 4.86 × 10^−4^ M FeCl_3_·6H_2_O, 4.40 × 10^−5^ M H_3_BO_3_, 9.24 × 10^−6^ M MnCl_2_·4H_2_O, 2.30 × 10^−6^ M ZnCl_2_, 5.29 × 10^−6^ M Na_2_MoO_4_·2H_2_O, 3.16 × 10^−7^ M CuSO_4_·5H_2_O, 1.68 × 10^−7^ M CoCl_2_·6H_2_O, and 9.00 × 10^−9^ M vitamin B12) was used (Włodarczyk et al., 2020). When necessary, kanamycin monosulfate (50 μg mL^−1^), gentamicin sulfate (40 μg mL^−1^), carbenicillin disodium salt (40 μg mL^−1^), or spectinomycin dihydrochloride (40 μg mL^−1^) was added to the culture. After inoculation to an optical density of 750 nm (OD_750_) = 1.0, cyanobacteria were cultured under continuous illumination with white fluorescent lamps at 100 μmol photons·m^−2^·s^−1^ (LL) or white light-emitting diodes at 500 μmol photons·m^−2^·s^−1^ (HL) with rotary shaking at 100 rpm (Kato et al., 2023). On day 2, isopropyl-β-d-thiogalactopyranoside (IPTG) was added to the KC156 and KC0157 cultures at a final concentration of 5 mM to induce heterologous gene expression.

**Table 1:** *Picosynechococcus* sp. strains used in this study.

Strain	Genotype
PCC 7002	Wild-type
KC0111	Δ*glpK*::P_*psbA2*_-*CgGDH*-*ScGLN1-*Km^R^
KC0112	KC0111 Δ*ldhA*::P_*trc*_-*EcLldD*-*EcLldP-*Gm^R^
KC0136	KC0111 Δ*glgC*::Amp^R^
KC0155	KC0111 Δ*mdh*::P_cLac143_-Sp^R^
KC0156	KC0111 Δ*mdh*::P_cLac143_-*CgPYC*^*P458S*^*-*Sp^R^
KC0157	KC0111 Δ*mdh*::P_cLac143_-*CgPYC*^*P458S*^-*CgCS-*Sp^R^

### 2.2 Construction of plasmids

All exogenous genes used in this study were codon-optimized using a gene synthesis service (Genscript Japan, Tokyo, Japan) and cloned into plasmids harboring the promoter and arms for homologous recombination using an In-Fusion HD Cloning Kit (Takara Bio USA, Mountain View, CA, USA). The plasmid pSKglpK-psbA2-CgGDH-ScGLN1 was used to introduce genes encoding GDH from *C. glutamicum* (CgGDH, WP_003856385.1) and GS from *S. cerevisiae* (ScGLN1, NP_015360.2) into the *glpK* pseudogene site of PCC 7002 (SYNPCC7002_A2842). *CgGDH* and *ScGLN1* were expressed under the control of the *psbA2* promoter (Fig. S1). The plasmid pSGldhA-trc-EcLldD-EcLldP, developed in a previous study, was used for deletion of the *ldhA* gene (SYNPCC7002_G0164) and expression of the *E. coli* genes encoding LldD (EcLldD, NP_418062.1) and LldP (EcLldP, NP_418060.1) using the constitutive *trc* promoter (Kato et al., 2023). The plasmid pSAglgC was used to delete the *glgC* gene (SYNPCC7002_A0095). The plasmid pSSmdh-cLac143 was used to delete the *mdh* gene (SYNPCC7002_A2093). The plasmids pSSmdh-cLac143-CgPYC and pSSmdh-cLac143-CgPYC-CgCS were constructed based on pSSmdh-cLac143 to introduce the *C. glutamicum* genes encoding PYC^P458S^ (CgPYC^P458S^, WP_011013816.1) and CS (CgCS, WP_011013914.1) into the *mdh* gene site. The *CgPYC*^*P458S*^ and *CgCS* genes were expressed under the IPTG-inducible cLac143 promoter (Markley et al., 2015). The DNA sequences of these genetic elements are listed in Table S1.

### 2.3 Transformation of cyanobacteria

Cyanobacteria were transformed as previously described (Kato et al., 2024). The cells were cultured in Medium A2 until they reached the mid-exponential phase (OD_750_ = approximately 1.0). The cells were washed once with the medium and resuspended in 0.1 vol of the medium. Following this, 1 μg of the aforementioned plasmid was added to the cell suspension (100 μL), and the mixture was slowly rotated in the dark overnight. The mixture was then placed under dim light conditions for 30 min and spread onto a 0.45-μm pore-size nitrocellulose filter (Merck Millipore, Burlington, MA, USA) on a Medium A2 agar plate containing 3 g L^−1^ Na_2_S_2_O_3_·5H_2_O. The agar plates were incubated at 30 °C for 2 days under continuous illumination with white fluorescent lamps. Subsequently, the nitrocellulose filter was transferred onto a fresh Medium A2 agar plate containing 3 g L^−1^ Na_2_S_2_O_3_·5H_2_O and the corresponding antibiotics for selection, and the agar plates were further incubated until colonies appeared. Single colonies were individually cultured in Medium A2, and integration and complete segregation were confirmed using direct PCR (Fig. S1 and S2). The DNA sequences of the primer pairs used in this study are listed in Table S2.

### 2.4 Metabolic analysis

To analyze metabolites released into the medium, clear supernatants without cells were obtained by centrifuging the culture broth at 8000 × *g* for 5 min. The supernatant (500 μL) was mixed with 500 μL of chloroform pre-cooled to 4 °C by vortexing. Following this, the mixture was centrifuged at 14,000 × *g* for 5 min at 4 °C, and the upper layer was filtered using an Amicon Ultra-0.5 Centrifugal Filter Unit UFC5003BK (Merck Millipore) by centrifugation at 14,000 × *g* at 4 °C. After adding l-methionine sulfone and piperazine-1,4-bis(2-ethanesulfonic acid) (PIPES) as internal standards at a final concentration of 400 μM each, the samples were subjected to capillary electrophoresis time-of-flight mass spectrometry (CE-TOF MS) using a G7100 CE and G6224AA liquid chromatography-mass selective detector (LC/MSD) TOF system (Agilent Technologies, Santa Clara, CA, USA) (Hasunuma et al., 2019; Kato et al., 2023).

To analyze intracellular metabolites, culture broth containing cells equivalent to 5 mg dry weight was immediately mixed with four volumes of 32.5% (*v/v*) pre-cooled methanol (−30 °C) by vortexing. The mixture was then centrifuged at 8000 × *g* for 3 min at 4 °C, and the supernatant was completely removed. Following this, 15 mL of 20 mM ammonium carbonate was added to the cell pellet, and the mixture was centrifuged again at 8000 × *g* for 3 min at 4 °C. After the supernatant was completely removed, the cell pellet was immediately resuspended in 1 mL of pre-cooled methanol (−30 °C) containing 37.5 μM l-methionine sulfone and 37.5 μM PIPES as internal standards. Next, 500 μL of the mixture was added with 200 μL of ultrapure water and 500 μL of chloroform pre-cooled to 4 °C and vortexed for 30 s. After centrifugation at 14,000 × *g* for 5 min at 4 °C, the aqueous layer was collected and filtered using a UFC5003BK filter unit (Merck Millipore), as described above. The resulting sample (300 μL) was then dried *in vacuo* using a CEV-3100 centrifugal evaporator (EYELA, Tokyo, Japan) and resuspended in 20 μL of ultrapure water before being subjected to CE-TOF MS (Hasunuma et al., 2019; Kato et al., 2023).

## 3 Results

### 3.1 Development of glutamine-producing cyanobacteria

A comprehensive understanding of amino acid assimilation capabilities in cyanobacteria is currently limited. The uptake of glutamate, a precursor of glutamine in the GS-GOGAT cycle, has been reported to be extremely low in PCC 6803 (Matsudaira et al., 2020). However, the glutamine uptake activity of cyanobacteria, including PCC 7002, remains unclear. To examine whether this cyanobacterial strain is suitable for glutamine production, the present study preliminarily examined glutamine consumption by PCC 7002 under phototrophic culture conditions. Glutamate supplemented in the culture medium of PCC 7002 was completely depleted by day 4 (Fig. S3), suggesting that the cyanobacteria consumed extracellular glutamate. Conversely, the decrease in the concentration of glutamine was substantially smaller than that observed for glutamate, despite the instability of glutamine in liquid media (Tritsch and Moore, 1962). This result suggested that the assimilation activity of extracellular glutamine is relatively low in PCC 7002, providing evidence that this cyanobacterial strain is suitable for glutamine production.

**Fig. 2:**
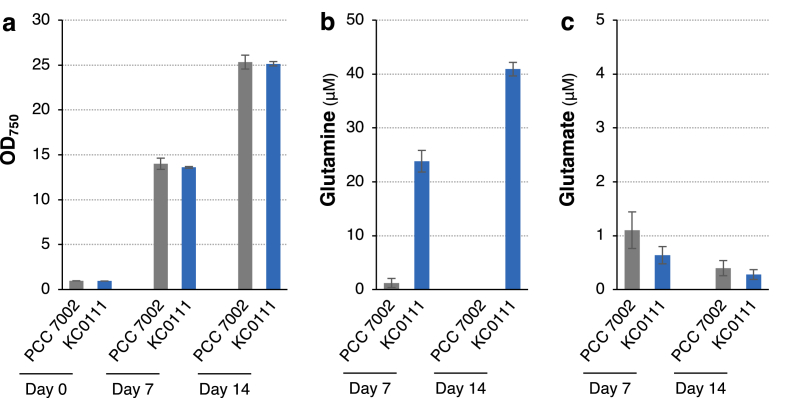
Evaluation of cyanobacteria harboring the *CgGDH* and *ScGLN1* genes. Wild-type (PCC 7002) and PCC 7002 with *CgGDH*/*ScGLN1* introduction (KC0111) were cultured photoautotrophically. (a) Optical density of the culture at 750 nm (OD_750_). (b) Glutamine concentration in the culture supernatant. (c) Glutamate concentration in the culture supernatant. Results represent the mean ± standard deviation of three replicate experiments.

Therefore, this study employed PCC 7002 as the host strain and examined valuable genetic engineering approaches to enhance glutamine production in cyanobacteria (Table 1 and Fig. S1). Previous studies on *C. glutamicum* have shown that enhancing GS activity is beneficial for improving glutamine production (Lv et al., 2021; Liu et al., 2021). Therefore, as a well-characterized enzyme whose function has been confirmed, the GS gene from *S*. *cerevisiae* (*ScGLN1*), was introduced into PCC 7002 to enhance glutamine synthesis from glutamate (Fig. 1a and b). Additionally, a gene encoding GDH from *C. glutamicum* (*CgGDH*), which was also suggested to be useful in a previous study (Fan et al., 2020), was introduced to enhance glutamine synthesis from 2-oxoglutarate. The resulting strain, KC0111, harboring the *CgGDH* and *ScGLN1* genes using the *psbA2* promoter, grew similarly to PCC 7002 under photoautotrophic conditions (Fig. 2a). The results revealed an increase in the glutamine concentration released in the culture supernatant in KC0111 (40.91 μM on day 14) compared to PCC 7002 (not detected on day 14) (Fig. 2b). Glutamate was also released in the culture supernatant, although the concentration was similar between PCC 7002 and KC0111 (Fig. 2c). Thus, the introduction of *CgGDH* and *ScGLN1* was shown to be useful for the development of glutamine-producing cyanobacteria.

### 3.2 Metabolic analysis for improving glutamine production

**Fig. 3:**
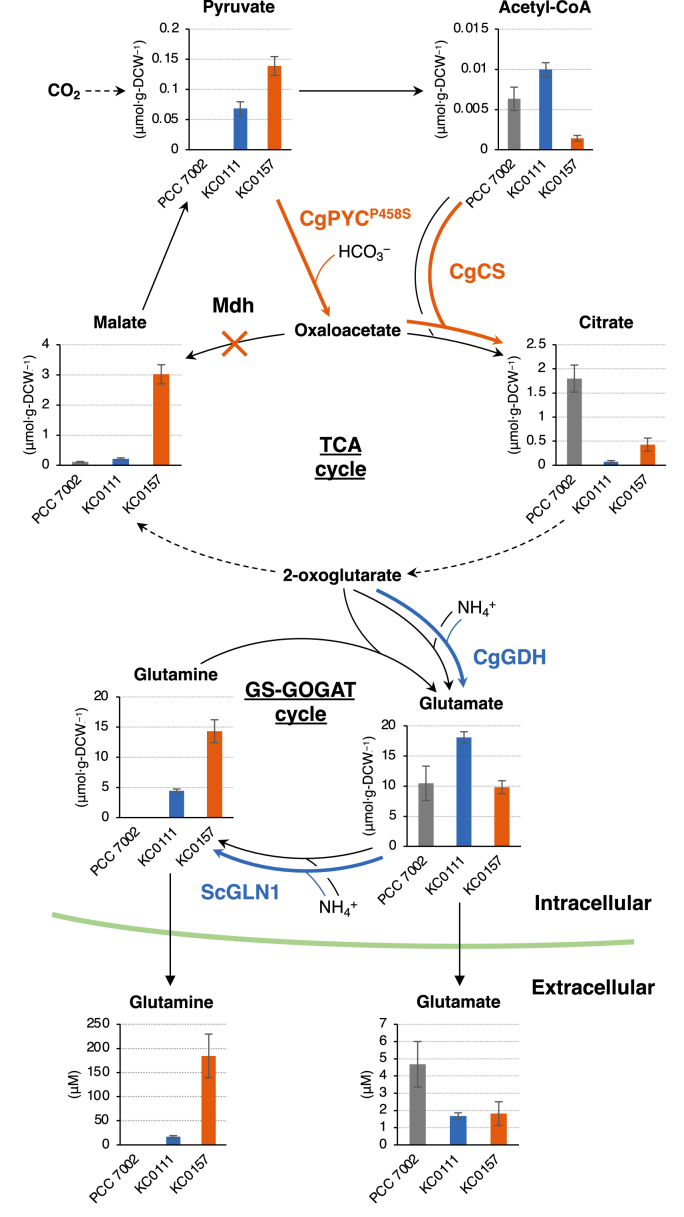
Metabolic analysis of glutamine-producing cyanobacteria. Wild-type (PCC 7002), PCC 7002 with CgGDH/ScGLN1 introduction (KC0111), and KC0111 with *mdh* deletion and *CgPYC*^*P458S*^/*CgCS* introduction (KC0157) were phototrophically cultured. For KC0157, 5 mM isopropyl-β-d-thiogalactopyranoside (IPTG) was added on day 2 of culture. The intracellular metabolites of the strains were comprehensively analyzed on day 7. Metabolic modifications in the pathway are illustrated based on those in KC0157. DCW: dry cell weight, GS-GOGAT cycle: glutamine synthase-glutamate synthase cycle, TCA cycle: tricarboxylic acid cycle. Results represent the mean ± standard deviation of three replicate experiments.

Metabolic analysis is a powerful tool for determining rate-limiting steps in synthetic pathways (Kato et al., 2024). To improve glutamine production in KC0111, the levels of intracellular metabolites in the cyanobacteria were comprehensively analyzed (Fig. 3 and Fig. S4). Intracellular glutamine was detected in the glutamine-producing strain KC0111, but not in PCC 7002. Intracellular glutamate levels were also higher in KC0111 than in PCC 7002. These results suggest that the synthesis of glutamate and glutamine from 2-oxoglutarate was successfully enhanced by introducing the *CgGDH* and *ScGLN1* genes. In contrast, the intracellular citrate level decreased 22.5-fold in KC0111 compared with that in PCC 7002, whereas the levels of upstream metabolites such as pyruvate and acetyl-CoA were not decreased in KC0111 compared to PCC 7002. These results suggest that citrate synthesis is a bottleneck for glutamine production in KC0111.

### 3.3 Improvement of glutamine production through citrate replenishment

**Fig. 4:**
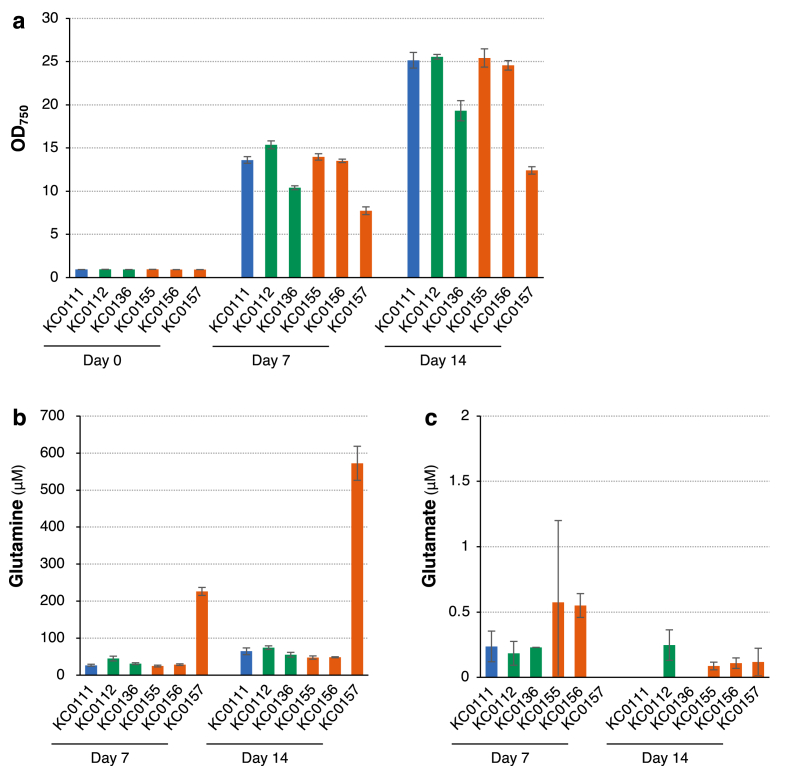
Enhancement of glutamine production in KC0111 through genetic engineering. PCC 7002 with *CgGDH/ScGLN1* introduction (KC0111) and its recombinants were cultured phototrophically. KC0112: *EcLldD/EcLldP*-introduced strain, KC0136: Δ*glgC* strain, KC0155: Δ*mdh* strain, KC0156: Δ*mdh* strain harboring *CgPYC*^*P458S*^, KC0157: Δ*mdh* strain harboring *CgPYC*^*P458S*^ and *CgCS*. For the KC0156 and KC0157 strains, 5 mM isopropyl-β-d-thiogalactopyranoside (IPTG) was added on day 2. (a) Optical density of the cyanobacterial cultures at 750 nm (OD_750_). (b) Glutamine concentration in the culture supernatant. (c) Glutamate concentration in the culture supernatant. Results represent the mean ± standard deviation of three replicate experiments.

Metabolic analysis of KC0111 suggested that glutamine production could be improved by enhancing citrate replenishment. A previous study reported that an l-lactate-assimilating cyanobacterial strain was developed in PCC 7002 by introducing the NAD-independent l-lactate dehydrogenase gene *lldD* (*EcLldD*) and the lactate permease gene *lldP* (*EcLldP*) from *E. coli* (Kato et al., 2023). During l-lactate assimilation, the intracellular citrate level increased 5.7-fold, likely because of the distributed metabolic flux from l-lactate, consequently increasing intracellular glutamine and glutamate levels. Additionally, glycogen deficiency caused by *glgC* deletion has been reported to enhance metabolic flux toward the TCA cycle in several cyanobacteria, including PCC 7002 (Davies et al., 2014; Jackson et al., 2015; Kato et al., 2024). Under nitrogen-abundant conditions, the Δ*glgC* strain of PCC 7942 extracellularly released distinct metabolites, including glutamine (Kato et al., 2024, 2025). The *de novo* synthesis ratios of 2-oxoglutarate, glutamine, and glutamate from CO_2_ increased in the Δ*glgC* strain, suggesting that synthesis of citrate, which is an intermediate of these metabolites, is also increased by glycogen deficiency. Based on these reports, the present study developed an l-lactate-assimilating strain (KC0112) and a glycogen-deficient strain (KC0136) using KC0111 as the parent strain to improve glutamine production (Fig. 1c and d). KC0112 cells cultured in the presence of l-lactate showed cell growth similar to that of KC0111 cells, whereas cell growth of KC0136 cells was lower than that of KC0111 cells (Fig. 4a). Glutamine production by KC0112 on day 14 was 74.33 μM, which was 1.15-fold higher than that of KC0111 (64.47 μM) (Fig. 4b). The KC0136 strain produced 55.17 μM glutamine on day 14, which was lower than that of the KC0111 strain. Glutamate production was comparable between strains (Fig. 4c). These results suggest that neither l-lactate assimilation nor glycogen deficiency significantly increased glutamine production.

To increase citrate synthesis in KC0111, direct metabolic engineering of the TCA cycle was examined. First, the *mdh* gene in KC0111 was deleted to prevent the conversion of oxaloacetate, as MDH in cyanobacteria has been suggested to catalyze the conversion of oxaloacetate into malate (Nakajima et al., 2014; Takeya et al., 2018; Katayama et al., 2022). The *mdh* deletion strain KC0155 showed similar cell growth and glutamine production to KC0111 (Fig. 4a and b). To replenish oxaloacetate, the mutated PYC gene from *C. glutamicum* (*CgPYC*^*P458S*^) was introduced with an IPTG-inducible cLac143 promoter (Ohnishi et al., 2002; Ikeda et al., 2006; Markley et al., 2015). The resulting strain, KC0156, showed similar cell growth and glutamine production to the KC0111 and KC0155 strains (Fig. 4a and b). To further examine whether the condensation reaction catalyzed by CS was a rate-limiting step in glutamine production, the CS gene from *C. glutamicum* (*CgCS*) was introduced along with *mdh* deletion and *CgPYC*^*P458S*^ introduction (Fig. 1e). Although the resulting strain, KC0157, showed slower cell growth than the KC0111, KC0155, and KC0156 strains, glutamine production was considerably improved (Fig. 4a and b). On day 14, glutamine production in the KC0157 strain reached 572.39 μM, which was 8.88-fold higher than that of the KC0111 strain. Metabolic analysis revealed that intracellular levels of citrate and glutamine in KC0157 cells were higher than those in KC0111 cells (Fig. 3). Acetyl-CoA levels notably decreased in KC0157 compared to PCC 7002 and KC0111, indicating that CS activity was increased by introduction of the *CgCS* gene. The levels of pyruvate and malate were higher in KC0157 than in PCC 7002 and KC0111, which may be because of increased supply of these metabolites through the TCA cycle as a result of metabolic modifications. These results are consistent with the possibility that citrate replenishment is a bottleneck in glutamine production. Furthermore, metabolic engineering to enhance the first step of the TCA cycle may increase glutamine production in cyanobacteria.

### 3.4 Optimization of culture conditions for glutamine production

**Fig. 5:**
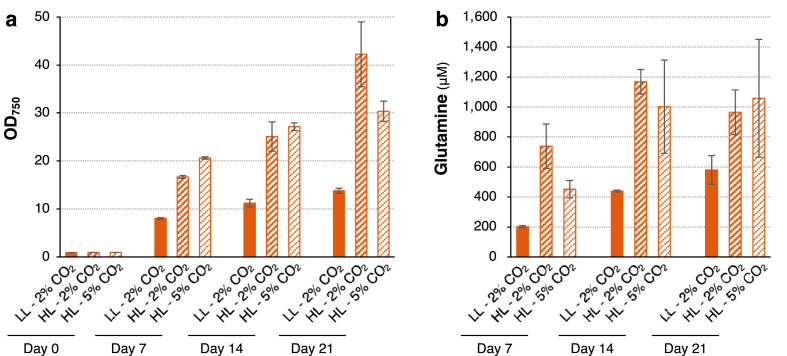
Cultivation of the KC0157 strain under different light intensities and CO_2_ concentrations. The KC0157 strain was cultured under illumination at 100 μmol photons·m^−2^·s^−1^ (low light, LL) or 500 μmol photons·m^−2^·s^−1^ (high light, HL) supplemented with 2% or 5% CO_2_. On day 2, isopropyl-β-d-thiogalactopyranoside (IPTG) was added to the culture to a final concentration of 5 mM. (a) Optical density at 750 nm (OD_750_) of the cyanobacterial cultures. (b) Glutamine concentration in the culture supernatant. Results represent the mean ± standard deviation of three replicate experiments.

In this study, culture conditions, including the medium, light intensity, and CO_2_ concentration, were optimized to increase glutamine production in KC0157. MAD2 medium, which contains 10.1-fold more nitrate and 5.53-fold more phosphate than Medium A2, was used to achieve high biomass production (Włodarczyk et al., 2020). When cultured in MAD2 medium under illumination at 100 μmol photons·m^−2^·s^−1^ (low light, LL) and CO_2_ supplementation at 2%, the cell growth of the KC0157 strain was similar to that observed in Medium A2 (Fig. 4a and 5a). When the light intensity was increased to 500 μmol photons·m^−2^·s^−1^ (high light, HL), growth of the KC0157 strain improved. Additionally, CO_2_ supplementation at 5% improved the growth of KC0157 cells. Glutamine production by KC0157 also improved under HL conditions (Fig. 4b), whereas glutamate was not detected extracellularly. Increasing the CO_2_ concentration to 5% did not result in higher glutamine production compared to 2% CO_2_. Thus, a maximum glutamine concentration of 1168.5 μM (170.76 mg L^−1^) was achieved using MAD2 medium under HL and 2% CO_2_ conditions after 14 days of cultivation.

## 4 Discussion

### 4.1 Glutamine production from CO_2_ and its extracellular release in cyanobacteria

This study aimed to achieve glutamine production in cyanobacteria using CO_2_ as the sole carbon source. Synthetic biology approaches were used to develop a glutamine-producing strain from PCC 7002, which originally released little extracellular glutamine (Fig. 1, Fig. 2b). A previous study reported that the heterogeneous expression of *CgGDH* increased theanine production in *E. coli*, showing that this gene from the natural glutamate producer, *C. glutamicum*, is useful for producing glutamate-derived compounds (Fan et al., 2020). ScGLN1 showed the highest activity among several GS enzymes from bacteria and yeast, including *C. glutamicum* (Lv et al., 2021). GOGAT activity was not enhanced in the present study because it catalyzes synthesis of the by-product glutamate (Liu et al., 2021). Therefore, the present study employed CgGDH and ScGLN1 as proven enzymes whose functions have been confirmed to enhance glutamine production. The resulting KC0111 strain showed increased glutamine concentration in the culture supernatant (Fig. 2b). The medium did not contain any organic carbon sources, indicating that KC0111 produces glutamine using atmospheric CO_2_ as its sole carbon source.

Bacterial GS enzymes are inactivated through reversible adenylation at high ammonium concentrations (Merrick and Edwards, 1995; Jakoby et al., 1999; Rehm et al., 2010). Knocking out the GS adenylyltransferase gene contributes to increased glutamine production in *E. coli* and *C. glutamicum* (Hayashi and Tabata, 2013; Zhu et al., 2020; Lv et al., 2021). Similarly, the mutagenesis of GS at its adenylylation site increases glutamine production in *C. glutamicum* (Liu et al., 2008). However, inactivation of GS through adenylylation has not been reported in cyanobacteria. Instead, GS activity in cyanobacteria is regulated by the binding of the GS-inactivating factors GifA and GifB (Bolay et al., 2018). The present study employed GS from *S. cerevisiae* (ScGLN1), which is not likely to be inactivated by GifA or GifB in cyanobacteria. In a previous study, the *glsA* and *glsB* genes encoding glutaminases were deleted to increase glutamine production in *E. coli* (Zhu et al., 2020). A glutaminase gene (slr2079) was previously identified in PCC 6803 (Zhou et al., 2008), whereas it is not conserved in the PCC 7002 strain used in this study. Another putative glutaminase gene, *purQ* (SYNPCC7002_A1545), was identified in PCC 7002 through a homology search. However, the present study did not investigate deletion of the *purQ* as its homolog in PCC 7942 (Synpcc7942_0819) has been identified as an essential gene (Rubin et al., 2015).

The cyanobacteria developed in this study released glutamine into the medium. The mechanism by which the recombinant strains released glutamine extracellularly remains completely unclear, and required further investigation. Previous studies have shown that the lysine exporter from *E. coli* is important for lysine secretion by cyanobacteria (Korosh et al., 2017; Dookeran and Nielsen, 2021). However, secretion of aromatic amino acids has been achieved in cyanobacteria without utilizing any heterogeneous exporters (Brey et al., 2020). In the present study, glutamine secretion was achieved without introducing any heterogeneous glutamine exporters (Fig. 2b). Possible candidates for native glutamine transporters include mechanosensitive channels (MSCs), which export amino acids, including glutamate, as osmolytes (Schleyer et al., 1993; Kawasaki and Martinac, 2020). In *C. glutamicum*, MscCG, which is encoded by the *NCgl1221* gene, plays a major role in glutamate secretion (Wang et al., 2018; Kawasaki and Martinac, 2020). However, in cyanobacteria, knowledge of MSCs is limited (Malcolm et al., 2012; Nanatani et al., 2013; Bachin et al., 2015). In PCC 7942, MscM (Synpcc7942_0610), a putative MSC, has been reported to contribute to the extracellular release of glutamate (Kato et al., 2025). Although their functionality has not been investigated, putative MSC genes have been identified in PCC 7002 through a homology search (such as SYNPCC7002_A2462, SYNPCC7002_A2580, and SYNPCC7002_A1177).

### 4.2 Increasing carbon flux through the TCA cycle for glutamine production

In this study, intracellular citrate levels in KC0111 were lower than those in PCC 7002 (Fig. 3), suggesting that citrate replenishment is a potential bottleneck in glutamine production. To enhance carbon flux through the TCA cycle, two metabolic engineering approaches, that is, assimilation of l-lactate and deficiency of glycogen synthesis, were examined before direct metabolic engineering of the TCA cycle (Kato et al., 2023, 2024; Davies et al., 2014; Jackson et al., 2015). However, neither l-lactate assimilation nor glycogen deficiency resulted in a substantial increase in glutamine production (Fig. 4b). In PCC 6803, MDH catalyzes the conversion of oxaloacetate to malate; in contrast, *mdh* has been reported as one of the missing genes in PCC 7942 (Nakajima et al., 2014; Takeya et al., 2018; Katayama et al., 2022; Rubin et al., 2015). Therefore, to increase carbon flux through the TCA cycle via direct metabolic engineering, the present study first deleted the *mdh* gene in the KC0111 strain. The deletion of *mdh* alone did not improve glutamine production (Fig. 4b). Next, *CgPYC*^*P458S*^ and *CgCS* genes from *C. glutamicum* were introduced to enhance citrate replenishment, as the native PYC gene was not detected in PCC 7002 cells. In *C. glutamicum*, PYC overexpression improves amino acid production through oxaloacetate replenishment (Peters-Wendisch et al., 2001; Xu et al., 2018). The present study employed *C. glutamicum* PYC with a P458S mutation (i.e., *CgPYC* ^*P458S*^) because this mutation has been reported to be beneficial for lysine production (Ohnishi et al., 2002; Ikeda et al., 2006). Previous studies have tested the utilization of CgPYC for photosynthetic lysine production in cyanobacteria. In PCC 7002, expression of CgPYC inhibited cell growth but increased specific lysine productivity at a high induction level (Wang et al., 2025). In UTEX 2973, introduction of the *CgPYC* gene alone was unsuccessful in improving lysine production (Dookeran and Nielsen, 2021). Similarly, glutamine production in the present study did not increase in KC0156 cells compared to that in KC0155 cells (Fig. 4b). Expression of *C. glutamicum* PEPC and *CgCS* improved succinate production in PCC 7942, and introduction of *CgCS* appeared to be effective (Lan and Wei, 2016). Additionally, previous studies have suggested that carbon flux through the TCA cycle is lower than that through glycolysis in PCC 6803, possibly because of the low CS activity in this strain (Young et al., 2011; Ito et al., 2019). These findings are consistent with the results of the present study, which showed that introduction of the *CgCS* gene significantly increased glutamine production (Fig. 4b). This supports the possibility that citrate replenishment is a bottleneck in the TCA cycle and in glutamine production, although further investigation is required to determine the usefulness of these metabolic modifications. Thus, based on metabolic analysis, the present study showed that engineering the gateway of the TCA cycle is beneficial for improving glutamine production in cyanobacteria.

### 4.3 Utility of photosynthetic glutamine production in cyanobacteria

The KC0157 strain developed in this study produced 1168.5 μM (170.76 mg L^−1^) glutamine (Fig. 5b). This is substantially lower than the titer of the fermentative glutamine producer *C. glutamicum* and is insufficient for practical applications (Lv et al., 2021; Liu et al., 2021). However, as the first proof-of-concept demonstration using cyanobacteria, the present study shows glutamine production directly using CO_2_ as the sole carbon source may be feasible in the future. Nitrogen-fixing cyanobacteria such as *Nostoc* (*Anabaena*) sp. PCC 7120 are ideal glutamine producers because they can directly utilize atmospheric CO_2_ and N_2_ as carbon and nitrogen sources, respectively (Ghosh et al., 2023; Haraguchi et al., 2025). In addition to photosynthetic production of glutamine and other nutrients, cyanobacteria can be used to treat waste compounds generated from animal cell cultures, such as l-lactate and ammonium (Kato et al., 2023; Chu et al., 2024; Haraguchi et al., 2024). Therefore, cyanobacteria are promising candidates for establishing a sustainable cell culture system that combines cultures of cyanobacteria and animal cells, i.e., a circular cell culture system, which could significantly contribute to animal cell culture-related industries in the future.

## Funding sources

This study was supported by the 10.13039/501100002770Cabinet Office, Government of Japan, 10.13039/501100020963Moonshot Research and Development Program. This study was supported by the Program for Forming Japan’s Peak Research Universities (J-PEAKS) from the 10.13039/501100001691Japan Society for the Promotion of Science (10.13039/501100001691JSPS).

## CRediT authorship contribution statement

**Yuichi Kato:** Conceptualization, Investigation, Methodology, Writing – original draft, Writing – review & editing. **Ayaka Tsuji:** Investigation, Writing – review & editing. **Yuji Haraguchi:** Writing – review & editing. **Tatsuya Shimizu:** Project administration, Writing – review & editing. **Akihiko Kondo:** Supervision. **Tomohisa Hasunuma:** Conceptualization, Project administration, Writing – review & editing.

## Declaration of competing interest

The authors declare that they have no known competing financial interests or personal relationships that could have appeared to influence the work reported in this paper.

## Data Availability

Data will be made available on request.
