# Supplementary material for: Photosynthetic production of glutamine through metabolic analysis-based engineering of Picosynechococcus sp. PCC 7002
Source: Metab Eng Commun. 2026 Jun 17;23:e00284. doi: 10.1016/j.mec.2026.e00284 (PMC13312541; doi:10.1016/j.mec.2026.e00284)
Supplement: Multimedia component 1 [file mmc1.docx]

**Table S1.** DNA sequences of genetic elements for cyanobacterial transformation.

| Name | Sequence (5′ to 3′) |
| --- | --- |
| Upstream region of the *glpK* site | TGAAGCGATTGGCTATGATCTACCAAAGCTGGCGATTTTTCTGCAAACCGAATTAGAACCACAATTTGATTTTTTAAAGCCGATTACCATTGCCAACCTGAAGGAAATAGCGATCGCCTTTATGGAGGATGGCCACGAGGCTTGTCGGGTCTCCCATTATTGTGGATCGGTACCGGATGAGCGGGCGAGCTTTAATTTACGCTTGCGGCAATATCGCCAGGCCCAGCCTTGGCTGCGAAATCATCTCGATCCTGCAAGGGGCGATCGCCTGCACCATTGGTCGGATCACCAACGCACCATTTTCTACGGCAGGCGCACCAATCCCGACACCCAGCAGCGGCTTGTGTTAGTGGCGCACATGGCCGGGGCTCCGAAGACCGTTGAGATTGGCAAATGGCTCGCCCTGGATTTGGATCGTTGGCAGTTGGCGATCGCCACACCGACTTTGAAGATCAACACCATCTATGACTTAGCCCAAATTCACTTGCACAATGGCGAAGGTTTTCTGTTATCTGAAATTCCTCCCTAAATGATGTCTTTTGAGCCTAAAAACACACTTTTTTGACCTAATTTAACCCATTTAAAAAA |
| Downstream region of the *glpK* site | TTACTGCTCCATGACCAACATTATTCCCTTGATCATGAAGCCTTTCTCTCAACCCTCAGCAACACAGAAAATTTACTCATTATTCAAGATCTAGATGGCGTTTGCATGGGGTTAGTCAAAGACCCCTTAACCCGCAAAATTGATCCTGACTATATCCGCGCCACACGCAAGTTTAGAGACCACTTCTTTGTCCTCACCAACGGTGAACATGAAGGCAGAAGGGGAGTAAATCGCATCGTTGAACGGGCATTTCGCAATGTTGAAGCCAAAGAGGAAACAAGCTATTTACCTGGTTTAGCAGCAGGGGGTGTGCAATGGCAGACAGATAATGGCCAAATTTCCCATCCCGGTGTTAGCCAAGCAGAACTCGATTTCCTTGCCACAGTGCCAGATTTAATTGGTCAAAGTTTAGGACAATTTTTTACTAAATATGTTGATATTTTTCCCGCTGAGCTTCAACCTGAGCTGATCCATGCTTCTGTTTTAGATAATCTCGTTTC |
| Upstream region of the *ldhA* site | AGACATTTCCCACAGACCACATCAAATTACAGCAATTGATCTAGCTGAAAGTTTAACCCACTTCCCCCCAGACCCAGAAGACCAGAGGCGCTTAAGCTTCCCCGAACAAACTCAACTGACCGAGGGGGAGGGAGCCGTAGCGGCGTTGGTGTTGGCGTAAATGACAGGCCGAGCAAAGAGCGATGAGATTTTCCCGACGATTGTCTTCGGGGATGTAATTTTTGTGGTGGACGCTTAAGGTTAAAACAGCCCGCAGGTGACGATCAATGCCTTTGACCTTCACATCCGACGGAATACAAACCAAGCCACAGAGTTCACAGCGCCAGTCTGCATCCTCTTTTACTTGTAAGGCGATCGCCTGCCAATCATCAGAATATCGAGAAGAATGTTTCATCTAAACCTAGCGCCGCAAGATAATCCTGAAATCGCTACAGTATTAAAAAATTCTGGCCAACATCACAGCCAATACTGTCTGTTTTGTGACCTATTTCGTGATTCTAACCCTAATTATTAAGAAATCACCAAAAAAACCATCTCCATTACCCCAAAGCACC |
| Downstream region of the *ldhA* site | AAACTGCGCCAAGAATAGCTCACTTCAAATCAGTCACGGTTTTGTTTAGGGCTTGTCTGGCGATTTTGGTGACATAGACAGTCACAGCAACAGTAGCCACAAAACCAAGAATCCGGATCGACCACTGGGCAATGGGGTTGGCGCTGGTGCTTTCTGTGCCGAGGGTCGCAAGATTTCCGGCCAGGGAGCCAATGTAGACATACATGATGGTGCCAGGGATCATCCCCACAGAGCCGAGGACATAGTCTTTTAGGGAAACGCCCGTGACCCCATAGGCATAGTTAAGCAGATTAAAGGGAAATACAGGTGAGAGACGCGTCAGGAGAACAATCTTCAGGCCTTCCTTGCCCACAGCTTCGTCGATGGCGCGAAATTTCGGGTTGTCGGCGATTTTTTGGCTCACCCATTGGCGGGCCAGATAACGACCCACTAGGAAAGCAGCGATCGCTCCTAGGGTTGCGCCAACAAAGACGTAAATTGATCC |
| Upstream region of the *glgC* site | CTGGAGTTGATCGACCACTTCTTCAANTCGGGCAAAATTACCCACGATGTCGATCCCTCCTTCCGTGGTGATTTCCTCGCGTTTTTCGGGGACGAGGGTCACGTAGTCGGGTTTGATGTCTAAGGCGATCGCCACCATTTCCGCCGTGGGGGCCATCTCTAAATTCAGGTGGGACTGCACCGTCTGCCGCAGAATTTTCACATCCCGGTCTTGGATATGCCGCCGGTCTTCCCGGAGGTGTACCGTAATCCCGTCGGCTCCCCCCAGTTCTGCTAAGGTTGCCGCCGCCACTGGATCTGGTTCTACCGTGCGTCGCGCTTGACGAATCGTCGCAACATGGTCAATATTCACACCGAGGGTCAGGCGAGGATTTGGGGAAACCATTAGGGAAAATAAGCTTAGAAGATAAAAAACTAAAAGCCCATTTTATCAGGGTTGCCAAAGGAGATGGAAGGGGCATTGCGCAGAATTTGCAGGGGATTACCACGATCCCCCACTGGATCTCTGGGGGCGGCGATCGCCCTTAAATAAACGATTCCCAAATCTTAAAATGACGTCGGTGAAAATCAGACCTATGATGGATTCTGGGGAAAAAATCCCGAAAAAGTTTGAATTGTTACTTTTCGAGATCCCCCCTCCAGTCATACACTTGACACAGAGGTCAAAGGCAGTCGTGAGGTCTTTTTTGTGAAACGAGTCCTAGGAATCATACTTGGCGGCGGCGCAGGTACTCGCCTATATCCGCTAACAAAACTCAGAGCTAAGCCCGCAGTACCTCTAGCAGGCAAATATCG |
| Downstream region of the *glgC* site | CAGAGCAGGCGAAAGAAAAGCCCTACATTGCGTCGATGGGGATCTACGTCTTTAAGAAACAAGTACTCCTCGATCTACTCAAAGAAGGCAAAGATAAAACCGATTTCGGGAAAGAAATTATTCCTGATGCGGCCAAGGACTACAACGTTCAGGCCTATCTCTTTGATGATTATTGGGCTGACATTGGGACCATCGAAGCGTTCTATGAAGCAAACCTTGGCTTGACGAAGCAGCCGATCCCACCCTTTAGTTTCTATGACGAAAAGGCTCCCATCTACACCCGGGCGCGCTACTTACCGCCGACGAAGGTGCTCAACGCTGACGTGACAGAATCGATGATCAGCGAAGGTTGCATCATTAAAAACTGCCGCATTCACCACTCAGTTCTTGGCATTCGCACCCGTGTCGAAGCGGACTGCACTATCGAAGATACGATGATCATGGGCGCAGATTATTATCAGCCCTATGAGAAGCGCCAGGATTGTCTCCGTCGTGGCAAGCCTCCCATTGGGATTGGTGAAGGGACAACGATTCGCCGGGCGATCATCGATAAAAATGCACGCATCGGTAAAAACGTGATGATCGTCAATAAGGAAAATGTGGAGGAGTCAAACCGTGAGGAGCTTGGCTACTACATTCGCAGCGGCATTACAGTGGTGCTAAAGAACGCCGTTATTCCCGACGGTACGGTCATTTAAGATCTGTTGCGGTTCTTGTTTTAAAAATTCTTTTTAAATCACATTTTTTGTCTAGGCAATGTGCCCTGTCTCTCGGTGAGATGGGGTTTTTTTGTGGACAGAGATAGCCTTTGGCATTTCTAAGCATCG |
| Upstream region of the *mdh* site | CTAACGAAGCCCAGGCCCTCGCCAATCCGCCCCTCTGGGCCCACATCAAAGTTTGTATGCGCCCCCTGCCCAATCAATTTTTCGATGGCTATGGCCTCTACCTCGAACAGGCCTACAGCTCCGATACCTCTGCCCCCTATCGTCTGCGCCTGTTTCACATCAAACCCGTCGATGACCACATGGAACTGGTGCATTACAAACCCAAAGATGACGCCAAAACCAAATATATGGGCGCTGCCCGCAACCCTGCCATGATGCAGCACTTTGACATGGCTGATCTAGACCCCATGCCCGGTTGCGACATGATCGTCACCTGGAGTGGGACGAGCTTCAAAGGCACTGTCCAGGCCGGAAAAGGCTGCCGGGTTGTGCGTTATAACAAAGAGAGTTACCTCGATAACAGCTTTGAAATCACCGATAACGCCTTGATCAGTATCGATCGCGGTCGTGATCCGGTCACCAATGAAATCCTCTGGGGCTCCCTGGCGGGGGCGTTTGAGTTTGAAAAGATCAACAATTTCTCTGGGGAAGTTCAACCCCATTAACCTCTAAAATAAAATTCATCACAATTAGGAGAGAAGCAAAAATTATGGCGGCTAAACTAAAGAAAGGAAGCCTTGTGCGGGTGATTAAAGAACAATTCACCAATAGCCTCGAAGCGAAAGCGAGTGATTCCCGTTTACCCGCCTACTTTTTCGGAAGCCAAGGCGAAATCCTCGATCTCGACGATGAATACGCCTTTGTGCGTTTCTATACCCCAACTCCCAGTGTTTGGCTCCGTCTCGATCAACTCGAAGCTGTTTAAATTTGGAATAGTCCC |
| Downstream region of the *mdh* site | TGCCCTGTCGGTTGGGGTGCCGGGGCGTAGAAGCAATTGTTGAGATCCCTTTGACCGATGCTGAACTGGCACAATTGCATCGCTCTGCGGCCTCGGTACGGGATAGCCTCGATAAAGCCCTCTCTAAACTGAGTCTTTAACAGATCGATCCATGGCAGTGTCGGTGGAATATTGGTTAATTTCCCTGGAGCAACTGGGACTGTCAACAAAGCTGTGGTGGCTCTTGAGCGACGACGAACAACAGCGGGCCAAGGAGTTTAAATTCCTAGATTTGCAACGGCGTTTTATTCTGGCACGGGCTGGTTTACGGCAAATCCTTGGGTTTTATCTCCAGCGAGATCCCAGGGCGATCGCCTTTGATTATGGTGACCACGGCAAACCGCTGTTGCCAGACATTGCGTTTAATTTATCCCACACACAAAAATTAGCCCTCTGCGCCATTAGCTTAGATGTTCCCCAAGCCCATTTAGGGGCGGATTTAGAAGCAAAAAGACGAAAAAGTGACATTCTGGGGCTCGCAAAACGATTTTTCACAGCGACAGAATCTAATTTCCTGCAACAGCTCCCAGAAACAGCGCAACAATCAGCCTTTTTCCAACTCTGGACAGCGAAGGAAGCTTATCTAAAGGGAATCGGCTGCGGTCTCCAAGGGGGCCTAGACCGCGTCGAAGTCCGCTTAACGCCGACGCCACAACTCCTGACGCCAGAGCAACAGCCTTGGTCATTATCGCTGTTTGCACCCAACGCTGATCATTGGGGGGCGATCGCCATTGATCAACCCGGTGCCCAGTTTACAAACCGGGGGGAATGGATGCTATATGATGGG |
| *psbA2* promoter | TCATTATTTCATCTCCATTGTCCCTGAAAATCAGTTGTGTCGCCCCTCTACACAGCCCAGAACTATGGTAAAGGCGCACGAAAAACCGCCAGGTAAACTCTTCTCAACCCCCAAAACGCCCTCTGTTTACCCATGGAAAAAACGACAATTACAAGAAAGTAAAACTTATGTCATCTATAAGCTTCGTGTATATTAACTTCCTGTTACAAAGCTTTACAAAACTCTCATTAATCCTTTAGACTAAGTTTAGTCAGTTCCAATCTGAACATCGACAAATACATAAGGAATTATAACC |
| *trc* promoter | TTGACAATTAATCATCCGGCTCGTATAATG |
| cLac143 promoter | CGAATTGTGAGCGCTCACAATTCGGAATTCTTAACAAAAAAGCAGGAATAAAATTAACAAGATGTAATTGACATAAGTCCCATCACCGTTGTATAAATGTGTGGAATTGTGAGCGGATAACAATTTCACACA |
| *rbcL* terminator | GTTACAGTTTTGGCAATTACTAAAAAACTGACTTCAATTCAATGTTAGCCCGCTCCCGCGGGTTTTTTGTTGCTTTTTCACAGTGACTATAGGTAATCAGCAACACAATACGGCCCTGTTCTTTGGACAGTTTTTGTATAATGTTGACCGCATCCTGACCGGATTTTTTATCTAAGTGGGGA |
| *lacI* cassette | TTTACAGCTAGCTCAGTCCTAGGTACAATGCTAGCTACTAGTGCATCGCTATCGCGTATACTATTTAGGAGACACATACCATGAAGCCGGTGACCCTCTACGATGTGGCGGAATACGCGGGTGTATCCTATCAGACCGTTTCCCGCGTGGTCAACCAAGCGTCTCATGTCAGTGCCAAAACGCGCGAAAAAGTCGAAGCAGCGATGGCTGAGCTGAACTACATCCCCAACCGTGTCGCGCAGCAGTTGGCAGGCAAGCAAAGCCTGCTGATTGGTGTGGCCACGAGTTCGTTGGCACTCCACGCCCCCAGCCAGATCGTGGCAGCGATCAAGTCGAGGGCCGATCAGTTAGGTGCCAGTGTTGTGGTGAGCATGGTGGAACGCAGTGGCGTTGAAGCGTGCAAAGCCGCGGTTCACAACCTGTTGGCTCAGCGGGTTTCCGGTTTGATTATTAACTATCCCTTGGATGACCAAGATGCCATCGCGGTGGAGGCGGCTTGTACCAATGTCCCTGCTCTGTTCCTGGATGTTAGCGATCAGACACCGATTAATAGTATCATTTTTAGCCACGAAGATGGCACCCGTCTCGGCGTTGAGCATCTGGTGGCGCTCGGTCATCAGCAAATCGCACTGCTCGCTGGACCACTCTCTAGTGTCTCTGCTCGACTGCGCCTCGCCGGTTGGCACAAGTATCTCACTCGCAATCAAATTCAGCCGATTGCTGAACGAGAAGGCGATTTTTCAGCTATGAGCGGCTTCCAACAGACGATGCAAATGCTTAATGAGGGCATCGTCCCCACTGCTATGCTGGTTGCCAACGATCAAATGGCCTTGGGGGCTATGCGGGCGATCACCGAGTCCGGGCTGCGAGTCGGGGCTGATATTTCAGTAGTCGGCTACGACGACACGGAAGACAGTAGCTGCTACATTCCGCCGCTCACAACGATCAAACAAGACTTTCGGCTACTTGGACAAACTTCGGTCGATCGCTTGCTGCAGCTAAGCCAAGGCCAAGCCGTGAAAGGCAATCAGCTTTTACCAGTCTCGCTAGTCAAGCGCAAAACCACGTTGGCACCCAATACCCAAACTGCAAGCCCTCGGGCCCTGGCCGACTCGCTGATGCAACTGGCACGCCAGGTGTCACGTCTAGAGTCGGGGCAGTAA |
| Km^R^ cassette | CCGGAATTGCCAGCTGGGGCGCCCTCTGGTAAGGTTGGGAAGCCCTGCAAAGTAAACTGGATGGCTTTCTTGCCGCCAAGGATCTGATGGCGCAGGGGATCAAGCTCTGATCAAGAGACAGGATGAGGATCGTTTCGCATGATTGAACAAGATGGATTGCACGCAGGTTCTCCGGCCGCTTGGGTGGAGAGGCTATTCGGCTATGACTGGGCACAACAGACAATCGGCTGCTCTGATGCCGCCGTGTTCCGGCTGTCAGCGCAGGGGCGCCCGGTTCTTTTTGTCAAGACCGACCTGTCCGGTGCCCTGAATGAACTGCAAGACGAGGCAGCGCGGCTATCGTGGCTGGCCACGACGGGCGTTCCTTGCGCAGCTGTGCTCGACGTTGTCACTGAAGCGGGAAGGGACTGGCTGCTATTGGGCGAAGTGCCGGGGCAGGATCTCCTGTCATCTCACCTTGCTCCTGCCGAGAAAGTATCCATCATGGCTGATGCAATGCGGCGGCTGCATACGCTTGATCCGGCTACCTGCCCATTCGACCACCAAGCGAAACATCGCATCGAGCGAGCACGTACTCGGATGGAAGCCGGTCTTGTCGATCAGGATGATCTGGACGAAGAGCATCAGGGGCTCGCGCCAGCCGAACTGTTCGCCAGGCTCAAGGCGAGCATGCCCGACGGCGAGGATCTCGTCGTGACCCATGGCGATGCCTGCTTGCCGAATATCATGGTGGAAAATGGCCGCTTTTCTGGATTCATCGACTGTGGCCGGCTGGGTGTGGCGGACCGCTATCAGGACATAGCGTTGGCTACCCGTGATATTGCTGAAGAGCTTGGCGGCGAATGGGCTGACCGCTTCCTCGTGCTTTACGGTATCGCCGCTCCCGATTCGCAGCGCATCGCCTTCTATCGCCTTCTTGACGAGTTCTTCTGA |
| Gm^R^ cassette | TTGACATAAGCCTGTTCGGTTCGTAAACTGTAATGCAAGTAGCGTATGCGCTCACGCAACTGGTCCAGAACCTTGACCGAACGCAGCGGTGGTAACGGCGCAGTGGCGGTTTTCATGGCTTGTTATGACTGTTTTTTTGTACAGTCTATGCCTCGGGCATCCAAGCAGCAAGCGCGTTACGCCGTGGGTCGATGTTTGATGTTATGGAGCAGCAACGATGTTACGCAGCAGCAACGATGTTACGCAGCAGGGCAGTCGCCCTAAAACAAAGTTAGGTGGCTCAAGTATGGGCATCATTCGCACATGTAGGCTCGGCCCTGACCAAGTCAAATCCATGCGGGCTGCTCTTGATCTTTTCGGTCGTGAGTTCGGAGACGTAGCCACCTACTCCCAACATCAGCCGGACTCCGATTACCTCGGGAACTTGCTCCGTAGTAAGACATTCATCGCGCTTGCTGCCTTCGACCAAGAAGCGGTTGTTGGCGCTCTCGCGGCTTACGTTCTGCCCAAGTTTGAGCAGCCGCGTAGTGAGATCTATATCTATGATCTCGCAGTCTCCGGCGAGCACCGGAGGCAGGGCATTGCCACCGCGCTCATCAATCTCCTCAAGCATGAGGCCAACGCGCTTGGTGCTTATGTGATCTACGTGCAAGCAGATTACGGTGACGATCCCGCAGTGGCTCTCTATACAAAGTTGGGCATACGGGAAGAAGTGATGCACTTTGATATCGACCCAAGTACCGCCACCTAA |
| Amp^R^ cassette | CGCGGAACCCCTATTTGTTTATTTTTCTAAATACATTCAAATATGTATCCGCTCATGAGACAATAACCCTGATAAATGCTTCAATAATATTGAAAAAGGAAGAGTATGAGTATTCAACATTTCCGTGTCGCCCTTATTCCCTTTTTTGCGGCATTTTGCCTTCCTGTTTTTGCTCACCCAGAAACGCTGGTGAAAGTAAAAGATGCTGAAGATCAGTTGGGTGCACGAGTGGGTTACATCGAACTGGATCTCAACAGCGGTAAGATCCTTGAGAGTTTTCGCCCCGAAGAACGTTTTCCAATGATGAGCACTTTTAAAGTTCTGCTATGTGGCGCGGTATTATCCCGTATTGACGCCGGGCAAGAGCAACTCGGTCGCCGCATACACTATTCTCAGAATGACTTGGTTGAGTACTCACCAGTCACAGAAAAGCATCTTACGGATGGCATGACAGTAAGAGAATTATGCAGTGCTGCCATAACCATGAGTGATAACACTGCGGCCAACTTACTTCTGACAACGATCGGAGGACCGAAGGAGCTAACCGCTTTTTTGCACAACATGGGGGATCATGTAACTCGCCTTGATCGTTGGGAACCGGAGCTGAATGAAGCCATACCAAACGACGAGCGTGACACCACGATGCCTGTAGCAATGGCAACAACGTTGCGCAAACTATTAACTGGCGAACTACTTACTCTAGCTTCCCGGCAACAATTAATAGACTGGATGGAGGCGGATAAAGTTGCAGGACCACTTCTGCGCTCGGCCCTTCCGGCTGGCTGGTTTATTGCTGATAAATCTGGAGCCGGTGAGCGTGGGTCTCGCGGTATCATTGCAGCACTGGGGCCAGATGGTAAGCCCTCCCGTATCGTAGTTATCTACACGACGGGGAGTCAGGCAACTATGGATGAACGAAATAGACAGATCGCTGAGATAGGTGCCTCACTGATTAAGCATTGGTAA |
| Sp^R^ cassette | GTGGCACTTTTCGGGGAAATGTGCGCGGAACCCCTATTTGTTTATTTTTCTAAATACATTCAAATATGTATCCGCTCATGAGACAATAACCCTGATAAATGCTTCAATAATATTGAAAAAGGAAGAGTATGCGTAGCCGTAATTGGAGCCGTACCCTGACCGAACGTAGCGGCGGTAATGGTGCGGTGGCGGTGTTTATGGCGTGCTATGATTGCTTTTTTGGCGTGCAGAGCATGCCGCGTGCGAGCAAACAGCAGGCGCGTTATGCGGTGGGCCGTTGCCTGATGCTGTGGAGCAGCAACGATGTGACCCAGCAGGGCAGCCGTCCGAAAACCAAACTGAACATTATGCGTGAAGCGGTGATTGCGGAAGTGAGCACCCAGCTGTCTGAAGTGGTGGGCGTGATTGAACGTCATCTGGAACCGACCCTGCTGGCCGTGCATCTGTATGGCAGCGCGGTGGATGGCGGCCTGAAACCGCATAGCGATATTGATCTGCTGGTGACCGTGACCGTGCGTCTGGATGAAACCACCCGTCGTGCGCTGATTAACGATCTGCTGGAAACCAGCGCGAGCCCGGGTGAAAGCGAAATTCTGCGTGCGGTGGAAGTGACCATCGTGGTGCATGATGACATTATTCCGTGGCGTTATCCGGCGAAACGTGAGCTGCAGTTTGGCGAATGGCAGCGTAACGATATTCTGGCCGGCATTTTTGAACCGGCGACCATTGATATCGATCTGGCCATTCTGCTGACCAAAGCGCGTGAACATAGCGTGGCGCTGGTTGGTCCGGCAGCGGAAGAACTGTTTGATCCGGTGCCGGAACAGGACCTGTTTGAAGCGCTGAACGAAACCCTGACCCTGTGGAACAGCCCGCCGGATTGGGCGGGTGATGAACGCAACGTGGTGCTGACCCTGAGCCGTATTTGGTATAGCGCGGTGACCGGCAAAATTGCGCCGAAAGATGTGGCGGCGGATTGGGCCATGGAACGTCTGCCGGCGCAGTATCAGCCGGTGATTCTGGAAGCGCGTCAGGCGTATCTGGGCCAGGAAGAAGATCGTCTGGCCAGCCGTGCGGATCAGCTGGAAGAATTTGTGCATTACGTGAAAGGCGAAATTACCAAAGTGGTGGGTAAATAATAACTGTCAGACCAAGTTTACTCATATATACTTTAGATTGATTTAAAACTTCATTTTTAATTTAAAAGGATCTAGGTGAAGATCCTTTTTGATAATCTCATGACCAAAATCCCTTAACGTGAGTTTTCGTTCCACTGAGCGTCAGACCCC |
| *CgGDH* | ATGACGGTTGATGAACAAGTGAGTAACTACTACGACATGCTGTTGAAGCGCAACGCAGGTGAACCTGAATTTCACCAGGCGGTGGCTGAGGTCTTGGAATCACTAAAAATTGTTTTAGAGAAGGATCCCCACTACGCCGACTATGGACTGATCCAACGGCTTTGTGAGCCAGAGCGCCAGCTCATCTTTCGTGTGCCTTGGGTCGATGACCAAGGCCAGGTTCATGTGAATCGAGGCTTTCGGGTTCAGTTCAATAGCGCACTAGGTCCCTACAAAGGTGGGCTCCGCTTCCACCCGTCTGTTAACCTCGGCATTGTCAAGTTCCTGGGTTTTGAGCAAATTTTCAAAAATTCCCTCACCGGCCTACCGATTGGTGGCGGCAAAGGGGGCTCGGACTTTGATCCCAAGGGCAAATCGGATTTAGAAATTATGCGCTTCTGTCAGAGCTTCATGACAGAACTGCATCGACACATTGGAGAATATAGGGATGTCCCGGCTGGCGACATCGGGGTTGGCGGACGGGAAATCGGCTACCTGTTTGGCCATTATCGCCGGATGGCGAATCAACATGAGTCAGGGGTGCTGACAGGTAAGGGTTTGACTTGGGGGGGTTCTTTGGTGCGGACGGAGGCTACTGGCTATGGCTGCGTGTACTTTGTCAGCGAGATGATCAAAGCCAAAGGCGAATCGATTTCCGGTCAGAAGATCATCGTTTCGGGTAGTGGCAACGTAGCAACGTATGCGATCGAAAAAGCGCAAGAGCTGGGCGCGACAGTGATTGGCTTCAGTGATTCGTCCGGCTGGGTGCATACGCCCAACGGTGTCGATGTGGCAAAGCTGCGCGAGATCAAGGAAGTACGTCGTGCGCGCGTCAGTGTCTATGCAGATGAGGTGGAAGGCGCCACTTATCACACCGATGGCAGCATTTGGGATCTCAAGTGCGATATTGCCCTACCCTGTGCCACCCAAAATGAGCTTAATGGCGAGAATGCTAAAACCCTGGCGGACAACGGTTGCCGATTCGTTGCTGAAGGAGCCAACATGCCGAGCACGCCTGAAGCTGTCGAAGTGTTCCGCGAACGCGACATACGCTTTGGTCCAGGCAAGGCTGCAAATGCCGGCGGGGTCGCAACCAGCGCTCTCGAAATGCAGCAGAACGCGAGTCGCGATTCTTGGAGCTTTGAATACACCGATGAGCGACTGCAAGTTATCATGAAAAACATCTTTAAGACCTGCGCGGAGACGGCTGCCGAATACGGCCACGAAAATGACTACGTCGTAGGGGCTAACATTGCCGGGTTTAAAAAAGTCGCCGATGCCATGTTGGCGCAAGGAGTGATCTAA |
| *ScGLN1* | ATGGCGGAGGCTAGCATCGAAAAAACCCAGATTCTGCAAAAATATCTCGAACTGGATCAGCGCGGCCGGATTATCGCAGAATATGTCTGGATTGATGGCACCGGGAACTTGCGATCGAAAGGCCGAACTCTCAAGAAAAGAATTACCTCGATCGATCAACTGCCGGAGTGGAACTTTGATGGAAGTTCTACAAATCAGGCACCAGGTCACGACTCCGATATCTATTTAAAACCAGTTGCCTACTATCCCGATCCGTTCCGTAGGGGAGACAACATTGTGGTGCTAGCGGCTTGCTACAACAACGATGGTACGCCCAACAAGTTCAATCACCGCCACGAAGCAGCCAAACTGTTTGCCGCTCACAAGGATGAAGAGATTTGGTTTGGGCTTGAGCAGGAATACACGCTGTTCGACATGTACGACGATGTCTATGGCTGGCCGAAGGGCGGCTATCCAGCGCCGCAAGGACCTTACTACTGCGGCGTGGGGGCAGGCAAAGTCTACGCGCGCGATATGATTGAAGCCCACTATCGCGCCTGCCTCTATGCTGGTTTGGAAATTAGTGGGATCAACGCTGAAGTGATGCCTAGTCAGTGGGAATTCCAAGTCGGTCCCTGCACTGGCATTGACATGGGCGATCAACTCTGGATGGCTCGCTATTTCTTGCATCGCGTCGCAGAAGAATTTGGCATCAAAATCAGTTTCCATCCCAAGCCGCTGAAAGGCGACTGGAATGGTGCTGGCTGTCACACCAATGTTAGCACCAAAGAAATGCGGCAGCCTGGTGGGATGAAGTACATCGAGCAAGCGATTGAGAAGCTATCCAAACGCCATGCCGAGCATATCAAGTTGTACGGCAGCGATAATGACATGCGCCTGACGGGCCGACATGAGACTGCGTCAATGACAGCCTTTTCCTCGGGGGTAGCAAACCGGGGAAGCTCAATCCGCATTCCCCGTAGCGTTGCCAAGGAAGGCTACGGTTACTTTGAAGATCGCCGTCCGGCCAGCAATATTGATCCCTACTTGGTGACGGGTATCATGTGTGAGACGGTTTGTGGCGCGATCGACAATGCCGATATGACCAAGGAGTTTGAGCGGGAATCGTCTTAA |
| *EcLldD* | ATGATCATCAGTGCTGCCTCTGACTATCGAGCAGCTGCTCAGCGAATTCTACCTCCCTTCCTGTTCCACTACATGGATGGTGGTGCCTACTCGGAGTACACCCTCCGCCGTAATGTCGAGGATCTGAGTGAAGTGGCGCTGCGGCAGCGGATTCTGAAGAACATGAGCGACCTCTCCTTGGAAACCACCCTGTTCAACGAAAAACTCTCGATGCCGGTTGCGCTAGCGCCAGTAGGTCTCTGTGGCATGTACGCCCGTCGCGGTGAGGTGCAAGCCGCCAAAGCCGCTGATGCCCATGGAATTCCATTTACTTTATCGACGGTGTCGGTCTGCCCGATTGAAGAAGTTGCTCCGGCGATCAAGCGACCAATGTGGTTTCAACTCTACGTCCTGCGCGATCGCGGCTTCATGCGGAATGCCCTTGAGCGGGCTAAAGCGGCTGGCTGCAGCACGCTGGTCTTCACGGTCGATATGCCTACTCCCGGCGCTCGCTATCGCGATGCCCACTCCGGGATGTCCGGCCCCAACGCAGCGATGCGCCGCTATCTCCAGGCAGTCACCCATCCCCAGTGGGCTTGGGATGTTGGCCTCAATGGACGTCCCCACGATCTAGGCAATATTTCAGCTTATCTGGGTAAGCCCACGGGTCTGGAGGACTACATCGGGTGGCTGGGTAACAACTTTGATCCCAGCATCAGCTGGAAGGATTTAGAGTGGATTCGTGACTTTTGGGACGGTCCGATGGTGATCAAAGGCATTCTCGATCCGGAAGACGCGCGTGACGCAGTTCGCTTTGGCGCCGATGGCATCGTGGTCAGCAATCATGGCGGTCGCCAACTGGATGGCGTGCTGTCATCTGCTCGAGCATTGCCTGCTATCGCTGATGCTGTCAAAGGCGATATCGCGATCTTGGCGGACAGTGGAATTCGCAATGGGCTTGATGTGGTTCGGATGATTGCCTTGGGAGCAGACACAGTGCTCTTGGGGCGCGCCTTTCTCTATGCCCTTGCGACTGCCGGGCAAGCGGGCGTTGCCAATTTGCTCAACCTGATTGAGAAAGAAATGAAGGTCGCCATGACCTTGACAGGTGCGAAAAGCATTTCTGAAATCACCCAAGATAGTTTGGTACAGGGGCTGGGCAAGGAACTGCCTGCGGCCTTAGCACCGATGGCCAAGGGCAACGCTGCATAA |
| *EcLldP* | ATGAATCTGTGGCAGCAAAACTACGACCCAGCAGGCAACATCTGGCTGTCATCGCTTATTGCGTCCTTGCCGATTCTGTTCTTCTTCTTTGCATTGATCAAACTGAAGCTGAAAGGCTACGTCGCGGCGAGTTGGACGGTGGCTATTGCTCTGGCAGTGGCGCTGCTCTTCTACAAAATGCCGGTCGCCAATGCCTTAGCGTCTGTTGTCTATGGCTTTTTTTACGGCCTTTGGCCAATTGCCTGGATTATCATTGCAGCGGTTTTTGTCTACAAGATTTCGGTCAAAACCGGGCAGTTTGATATCATTCGATCGTCGATATTGAGTATCACCCCAGATCAGCGTCTCCAAATGCTGATTGTGGGCTTCTGCTTTGGGGCTTTCCTCGAAGGGGCAGCAGGGTTTGGAGCACCCGTGGCGATTACTGCTGCGTTGCTGGTCGGGCTGGGTTTTAAGCCTCTCTATGCAGCCGGACTCTGCTTGATCGTCAATACTGCCCCCGTTGCCTTTGGTGCGATGGGCATTCCCATCTTGGTCGCTGGTCAAGTCACGGGAATCGACAGTTTTGAGATTGGTCAGATGGTGGGGCGGCAACTCCCTTTCATGACGATCATCGTTCTGTTTTGGATCATGGCGATTATGGATGGCTGGCGAGGTATCAAGGAAACCTGGCCGGCGGTAGTGGTGGCCGGCGGGAGCTTCGCGATCGCCCAGTATCTCAGCAGCAACTTTATCGGTCCTGAACTGCCCGATATTATTTCTAGTCTGGTTAGCTTGCTTTGTCTGACTCTGTTCCTCAAACGCTGGCAACCGGTTCGTGTCTTCCGCTTTGGCGATTTGGGAGCCAGCCAGGTTGATATGACCCTAGCCCATACGGGTTATACCGCGGGCCAAGTGCTGCGCGCTTGGACCCCCTTCCTCTTTCTCACGGCCACCGTAACGCTCTGGAGCATACCGCCTTTCAAAGCACTGTTTGCCTCGGGAGGTGCCCTCTACGAGTGGGTGATTAATATTCCGGTGCCCTATCTTGACAAGTTGGTGGCACGGATGCCCCCAGTTGTCAGCGAAGCCACAGCCTACGCCGCAGTGTTCAAGTTTGACTGGTTTTCAGCCACGGGAACAGCCATTCTTTTTGCTGCTCTTCTCAGTATTGTTTGGCTCAAGATGAAACCGAGCGATGCGATCAGTACCTTCGGCTCTACGCTAAAAGAGTTAGCTCTACCGATCTACAGCATTGGGATGGTCCTAGCATTTGCCTTTATCAGCAATTATTCAGGCCTGTCTTCAACACTGGCCTTGGCTTTGGCCCATACAGGCCACGCGTTCACCTTTTTCTCGCCCTTTTTAGGTTGGCTGGGCGTCTTTCTGACCGGTTCCGACACCAGCTCCAACGCTTTGTTCGCGGCACTGCAGGCTACTGCAGCCCAACAGATTGGTGTCAGTGATTTGCTGTTGGTGGCTGCTAACACGACCGGCGGCGTGACTGGCAAGATGATCTCCCCCCAGTCGATCGCAATCGCTTGTGCTGCTGTGGGCTTAGTTGGCAAAGAAAGCGATTTGTTTCGCTTCACGGTCAAGCACTCCCTGATCTTCACCTGCATCGTTGGTGTGATCACCACACTACAAGCCTATGTGTTGACTTGGATGATTCCTTAA |
| *CgPYC^P458S^* | ATGAGCACTCACACATCAAGCACGCTTCCTGCTTTCAAAAAAATTTTGGTAGCCAATCGAGGCGAGATCGCTGTGCGCGCATTCCGTGCTGCTTTGGAAACCGGCGCGGCGACTGTGGCAATCTACCCCCGCGAGGATCGCGGTTCCTTCCATCGGTCCTTTGCCAGCGAAGCAGTGCGAATTGGCACGGAAGGCTCGCCCGTGAAAGCCTATCTCGACATTGACGAGATCATCGGCGCTGCAAAGAAAGTTAAAGCGGATGCGATCTATCCTGGGTATGGCTTTTTAAGTGAAAATGCGCAGCTGGCTCGGGAATGTGCTGAAAACGGCATCACCTTTATTGGACCAACCCCTGAGGTGCTGGATTTGACGGGTGATAAAAGCCGAGCCGTAACAGCTGCCAAAAAGGCCGGCCTGCCTGTCTTAGCTGAAAGTACGCCTTCGAAAAATATTGATGAAATTGTGAAGTCTGCTGAGGGCCAGACCTATCCGATTTTTGTCAAAGCAGTCGCCGGGGGGGGCGGTCGGGGGATGCGATTCGTGGCATCCCCAGACGAGCTGCGTAAGCTGGCCACAGAGGCCTCCCGAGAGGCGGAAGCCGCGTTTGGCGATGGAGCGGTCTATGTCGAACGCGCGGTGATCAACCCCCAACACATCGAAGTGCAGATTTTGGGAGATCACACGGGGGAAGTCGTTCATCTTTACGAACGAGACTGCTCGCTGCAACGGCGCCATCAAAAAGTTGTCGAAATCGCGCCGGCGCAGCACTTAGACCCGGAATTGCGGGATCGCATTTGTGCCGATGCCGTTAAGTTCTGCCGCTCGATCGGCTATCAAGGAGCAGGAACGGTTGAGTTCTTGGTCGATGAGAAAGGTAACCACGTTTTTATCGAGATGAATCCGCGCATTCAGGTCGAGCATACAGTTACGGAGGAAGTGACTGAAGTCGACCTCGTGAAGGCCCAGATGCGCTTGGCAGCGGGTGCCACTCTGAAGGAACTCGGACTGACCCAAGATAAGATTAAGACCCACGGCGCAGCCCTCCAGTGCCGCATAACCACAGAAGACCCCAACAATGGCTTTCGTCCCGACACAGGCACGATTACGGCTTATCGCTCCCCGGGCGGCGCGGGGGTCCGCCTGGATGGCGCCGCACAATTAGGTGGTGAAATCACGGCGCATTTTGATTCGATGCTCGTCAAAATGACCTGTCGGGGTAGCGACTTCGAGACAGCCGTGGCTCGCGCCCAGCGCGCACTCGCAGAGTTTACCGTCAGCGGTGTTGCTACGAATATCGGTTTCCTGCGAGCCCTCCTAAGGGAAGAAGATTTTACCAGCAAGCGCATTGCTACTGGTTTCATTGCAGACCATAGTCACCTACTGCAGGCCCCGCCAGCCGATGACGAGCAGGGCCGCATCCTAGACTATTTGGCGGATGTCACCGTGAACAAACCCCACGGCGTTAGGCCAAAAGATGTTGCGGCTCCGATCGACAAGCTGCCCAACATCAAGGATCTCCCACTGCCCCGCGGCAGTCGTGATCGACTAAAGCAACTGGGGCCGGCTGCCTTTGCGCGCGATCTGCGCGAGCAAGACGCTTTGGCCGTGACTGATACCACCTTCCGCGATGCCCATCAGTCGCTGCTTGCGACACGCGTGCGGAGCTTTGCACTGAAACCAGCGGCCGAAGCGGTGGCCAAGCTAACGCCTGAACTCCTTTCCGTTGAAGCTTGGGGCGGCGCAACTTACGACGTGGCCATGCGCTTTTTGTTTGAGGATCCTTGGGATAGATTAGATGAGCTCCGTGAGGCAATGCCAAATGTTAACATTCAAATGCTGCTGCGGGGCCGTAACACTGTGGGCTACACCCCCTATCCTGACAGCGTCTGCCGGGCGTTTGTAAAAGAAGCAGCCTCTTCTGGTGTTGACATCTTCCGGATCTTTGATGCGCTCAACGACGTGTCTCAGATGCGGCCGGCCATCGATGCCGTCCTGGAGACCAACACTGCCGTAGCCGAAGTGGCCATGGCCTACAGCGGTGACCTGAGCGATCCCAACGAGAAGCTCTATACGCTTGACTACTATCTCAAGATGGCGGAAGAAATTGTGAAAAGTGGCGCTCACATTCTGGCCATCAAAGACATGGCTGGTCTGCTCCGGCCAGCGGCAGTTACTAAGTTGGTCACCGCGTTGCGTCGCGAATTTGACCTCCCGGTCCATGTCCACACCCATGATACAGCCGGCGGACAGTTAGCTACCTACTTCGCCGCAGCGCAAGCGGGTGCTGATGCCGTCGACGGGGCTTCAGCTCCCCTCTCCGGGACTACGAGCCAACCCAGCCTCAGTGCCATCGTTGCTGCTTTTGCGCATACCCGCCGCGATACGGGTCTGTCGTTGGAGGCCGTATCGGATCTGGAACCCTACTGGGAAGCTGTACGGGGTCTCTACCTTCCCTTTGAGTCGGGTACCCCCGGCCCCACCGGGCGCGTGTACCGGCATGAGATTCCCGGAGGTCAGCTCAGCAATCTCCGCGCCCAAGCAACCGCTTTGGGCCTCGCTGATCGCTTTGAATTGATTGAGGATAACTACGCCGCTGTTAACGAAATGCTGGGTCGCCCTACTAAGGTTACACCTTCGTCAAAGGTCGTGGGCGATTTGGCGCTGCATTTAGTTGGGGCCGGTGTTGATCCAGCCGATTTTGCTGCGGATCCGCAAAAGTACGATATCCCGGACAGTGTCATTGCCTTCCTCCGTGGCGAACTGGGCAATCCCCCAGGCGGCTGGCCCGAGCCTTTGCGGACCCGAGCACTAGAAGGGCGGTCAGAAGGCAAAGCACCACTCACTGAGGTCCCTGAGGAGGAACAAGCCCACCTTGATGCCGATGATTCTAAAGAGCGTCGCAACTCGCTCAATCGCTTGCTGTTTCCCAAACCGACGGAGGAGTTTCTCGAACACCGTCGCCGCTTCGGAAATACCAGTGCTCTGGATGATCGAGAATTCTTCTACGGCTTGGTGGAGGGGAGAGAAACGCTGATTCGGCTGCCGGATGTGCGCACCCCGCTGCTAGTCCGACTAGATGCGATCTCCGAACCGGATGACAAAGGCATGCGCAATGTGGTGGCAAATGTCAATGGACAAATTCGCCCGATGCGGGTTCGCGATCGCAGCGTGGAAAGCGTTACCGCGACGGCGGAGAAAGCGGACAGTTCAAACAAGGGCCACGTCGCAGCGCCCTTCGCAGGCGTGGTGACGGTCACCGTTGCAGAAGGGGATGAAGTCAAGGCGGGCGATGCTGTAGCCATCATTGAAGCGATGAAGATGGAAGCTACGATCACCGCCAGTGTGGACGGTAAAATCGATCGCGTTGTTGTCCCGGCAGCAACTAAAGTGGAAGGTGGCGATCTAATTGTAGTCGTCAGTTAA |
| *CgCS* | ATGTTTGAGCGCGATATCGTGGCCACTGACAACAACAAGGCGGTTTTACACTATCCAGGGGGTGAATTTGAAATGGATATTATTGAAGCCTCCGAAGGCAATAATGGTGTTGTACTCGGCAAGATGCTCAGTGAAACAGGGCTTATCACTTTTGATCCAGGCTACGTGTCTACTGGGAGTACAGAAAGCAAAATCACGTACATCGATGGTGACGCGGGCATTTTGCGCTATCGCGGCTACGACATTGCAGACCTCGCAGAAAACGCCACCTTCAATGAAGTCAGCTATCTGTTGATCAACGGTGAGCTACCAACGCCAGATGAACTGCACAAGTTTAATGACGAGATTCGCCATCACACCCTGCTAGATGAAGATTTCAAAAGTCAGTTCAACGTGTTTCCTCGAGATGCCCATCCCATGGCTACCTTAGCCAGCTCGGTCAACATCCTGTCGACCTACTACCAAGATCAGCTCAATCCCTTGGATGAGGCACAACTAGACAAAGCAACCGTGCGTCTGATGGCCAAAGTGCCGATGCTCGCAGCCTATGCGCATCGCGCCCGCAAAGGCGCCCCCTACATGTATCCCGATAACTCCTTGAACGCTCGGGAGAACTTCCTTCGTATGATGTTCGGCTATCCGACCGAACCTTACGAGATTGATCCGATCATGGTCAAAGCTCTGGACAAGCTGCTGATTCTGCATGCCGATCACGAGCAGAACTGCTCAACGTCCACAGTGCGGATGATTGGCAGTGCTCAGGCCAACATGTTTGTGAGCATTGCAGGCGGGATCAATGCCCTCAGCGGTCCGTTGCATGGTGGTGCGAATCAAGCTGTCCTGGAGATGTTAGAAGACATCAAGTCGAATCACGGCGGCGATGCGACTGAGTTTATGAATAAGGTTAAAAATAAAGAGGATGGCGTAAGATTGATGGGTTTTGGCCACCGCGTTTATAAAAATTACGATCCCCGTGCTGCGATTGTCAAGGAAACCGCTCATGAGATTCTTGAGCATTTGGGAGGAGACGATCTGCTCGATTTGGCGATCAAACTCGAAGAGATCGCGCTCGCTGATGACTACTTCATCTCTCGCAAGCTCTACCCCAATGTCGATTTTTATACGGGTCTGATCTATCGGGCGATGGGCTTTCCGACGGACTTCTTCACGGTCCTGTTTGCGATCGGTCGCTTGCCGGGATGGATTGCCCACTACCGAGAGCAACTGGGGGCAGCTGGAAACAAAATTAATCGGCCTCGCCAGGTCTACACCGGCAACGAATCGCGGAAGCTGGTTCCCAGGGAAGAACGATAA |

**Table S2.** DNA sequences of the primer pairs used for segregation check.

| Site | Forward primer (5′ to 3′) | Reverse primer (5′ to 3′) |
| --- | --- | --- |
| *glpK* | GCGATCGCCTTTATGGAGGA | GTGTGGCGCGGATATAGTCA |
| *ldhA* | CTATGACATGATTACGAATTCAGACATTTCCCACAGACCACATCAAATTA | CTGCAGGTCGACGGATCCCCGGGGGATCAATTTACGTCTTTGTTGGCGCA |
| *glgC* | CGGGCAAAATTACCCACGATGTC | CGATGCTTAGAAATGCCAAAGGCTATCTC |
| *mdh* | ATGGAACTGGTGCATTACAAACCCAAAGAT | GGGAGCTGTTGCAGGAAATTAGATTCTGTC |

**Fig. S1** Schematic diagrams of the genome/plasmid regions of (a) *glpK* (SYNPCC7002_A2842), (b) *ldhA* (SYNPCC7002_G0164), (c) *glgC* (SYNPCC7002_A0095), and (d) *mdh* (SYNPCC7002_A2093) gene sites in the wild-type PCC 7002 strain and its recombinant strains. The upstream/downstream black boxes indicate homologous sequences used to introduce these genetic elements into the genome/plasmid regions. The PCR primer pairs used for segregation check are indicated by facing arrows.

**Fig. S2** Confirmation of complete segregation in the recombinant strains. The genome/plasmid regions of (a) *glpK*, (b) *ldhA*, (c) *glgC*, and (d) *mdh* genes were amplified through direct PCR from recombinant cells. The arrows indicate unmodified DNA fragments.

**Fig. S3** Consumption of glutamine and glutamate supplemented in the medium. PCC 7002 was phototrophically cultured in Medium A2 containing glutamine or glutamate, and their concentrations in the culture supernatant were monitored. Results represent the mean ± standard deviation of three replicate experiments.

**Fig. S4** Levels of intracellular metabolites in glutamine-producing strains. Wild-type (PCC 7002), PCC 7002 with the CgGDH/ScGLN1 introduction (KC0111), and KC0111 with *mdh* deletion and *CgPYC^P458S^*/*CgCS* introduction (KC0157) were phototrophically cultured. For KC0157, 5 mM isopropyl-β-d-thiogalactopyranoside (IPTG) was added on day 2 of culture. Intracellular metabolites of the strains were comprehensively analyzed on day 7. DCW: dry cell weight, GS-GOGAT cycle: glutamine synthase-glutamate synthase cycle, TCA cycle: tricarboxylic acid cycle. Results represent the mean ± standard deviation of three replicate experiments.
